# Anisakiasis Annual Incidence and Causative Species, Japan, 2018–2019

**DOI:** 10.3201/eid2810.220627

**Published:** 2022-10

**Authors:** Hiromu Sugiyama, Mitsuko Shiroyama, Ikuyo Yamamoto, Takashi Ishikawa, Yasuyuki Morishima

**Affiliations:** National Institute of Infectious Diseases, Tokyo, Japan (H. Sugiyama, I. Yamamoto, Y. Morishima);; Azabu University, Kanagawa, Japan (M. Shiroyama); BML, Inc., Saitama, Japan (T. Ishikawa)

**Keywords:** parasites, food safety, enteric infections, zoonoses, anisakis, anisakiasis, foodborne diseases, parasitic diseases, asymptomatic infections, universal health insurance, PCR, urticaria, seafood, Japan

## Abstract

Using data from 2018–2019 health insurance claims, we estimated the average annual incidence of anisakiasis in Japan to be 19,737 cases. Molecular identification of larvae revealed that most (88.4%) patients were infected with the species *Anisakis simplex* sensu stricto. Further insights into the pathogenesis of various anisakiasis forms are needed.

Anisakiasis is a foodborne zoonosis caused by ingestion of raw or undercooked fish and cephalopods parasitized by anisakid nematode larvae. As seafood consumption has increased globally (*1*), the incidence of anisakiasis has also increased worldwide (*2*,*3*). The population of Japan traditionally consumes large quantities of seafood (*4*), and consuming raw seafood, such as sushi and sashimi, is common. 

In response to the large number of annual cases, the government of Japan added anisakiasis under food poisoning in its Ordinance for Enforcement of the Food Sanitation Act to strengthen countermeasures in 1999, when food poisoning statistics included a case of anisakiasis (*5*). In 2012, the government amended the ordinance and registered anisakid nematodes, comprising *Anisakis* spp. and *Pseudoterranova* spp., as a single disease agent, *Anisakis*, in the list of food poisoning agents (*6*). These measures enabled the government to aggregate the number of *Anisakis* food poisoning cases, which showed a near-constant increase. However, epidemiologic studies have indicated that the number of food poisoning cases in Japan is considerably higher than those officially reported (*7*). For anisakiasis, the discrepancy between food poisoning statistics and the actual incidence is unclear because no nationwide investigation into anisakiasis incidence has been conducted since the 2012 amendment. 

We analyzed a large database of health insurance claim data (*8*) to estimate the national incidence of anisakiasis and determine the degree of discrepancy between foodborne statistics and actual incidence. Furthermore, we performed molecular identification of anisakid larvae isolated from infected patients to clarify the causative agent and adduce reasons for the high incidence of anisakiasis in Japan.

## The Study

To estimate the number of anisakiasis patients, we used an anonymized health insurance claim database from JMDC, Inc. (https://www.jmdc.co.jp), a commercial medical database provider in Japan. The nation has a universal health insurance system wherein medical institutions prepare health insurance claims that list the name of the disease or injury on a service basis. Medical expenses, other than a portion directly paid by patients, are reimbursed from taxes and mandatory insurance fees. In this process, medical institutions first submit health insurance claims to a specialized organization that assesses the appropriateness of treatment and amount of reimbursement. In this study, we used a database covering ≈8.43 million persons annually, accounting for >6% of the total population of Japan, during January 2018–December 2019. We extracted health insurance claims with the diagnosis code B81.0 in the International Classification of Diseases, 10th Revision (ICD-10), indicating *Anisakis* and *Pseudoterranova* infections.

The number of patients with anisakiasis registered in the target database was 991 in 2018 and 766 in 2019. However, the data revealed a male-biased sex ratio and a workforce-biased distribution for persons 20–39 years of age. Therefore, we adjusted the value in each group for estimation by stratifying the population of Japan by sex and age by using a previously described expanded estimate method (*9*), and data from the national census conducted by the Ministry of Internal Affairs and Communications in 2015 (https://www.stat.go.jp/english/data/kokusei/2015/final_en/final_en.html). On the basis of our adjustment, we estimate the number of patients in Japan with anisakiasis was 21,511 in 2018 and 17,962 in 2019. The number of patients with anisakiasis recorded in the food poisoning statistics during the same period was considerably lower, <1/40, in our estimation (Table 1).

**Table 1:** Reported and estimated number of patients with anisakiasis, Japan, 2018–2019

Year	Food poisoning cases*	Health insurance claims	
		No. in database†	Nationwide estimates‡
2018	478	991	21,511
2019	336	766	17,962
Average	407	878.5	19,737

*Data collected from Ministry of Health, Labour and Welfare. †Data extracted from JMDC Inc. (https://www.jmdc.co.jp) health insurance claim database, which covers ≈8.43 million persons annually. ‡Estimates calculated by stratifying the population of Japan by sex and age, as previously described (*9*).

**Figure 1:**
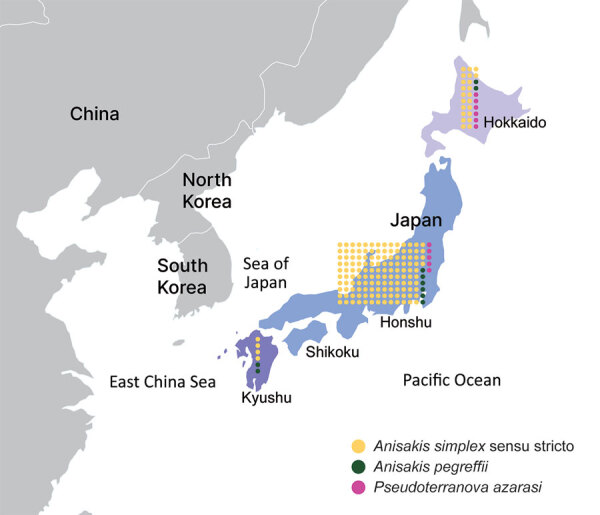
Anisakiasis patient number and causative species analyzed in study of anisakiasis in Japan, 2018–2019. Three geographic locations in Japan are noted: Hokkaido Island (North Japan), Honshu and Shikoku Islands (Central Japan), and Kyushu Island (Southwest Japan). Each dot indicates 1 patient, and color indicates the anisakiasis-causing species. Identifications were made at the sibling species level by using PCR and sequencing.

For molecular identification, we obtained 189 larvae of anisakid nematodes from BML, Inc. (http://www.bml.co.jp/eng), a privately-owned clinical testing laboratory in Japan. The larvae were isolated from 181 anisakiasis patients in 30 of 47 prefectures in Japan during 2018–2019 (Figure 1). We extracted DNA samples from individual larvae, used PCR to amplify the internal transcribed spacer 1 region, and sequenced the amplicons to distinguish between nematode genera and identify the *Anisakis* species. For *Pseudoterranova* larvae, we further amplified a portion of the NADH dehydrogenase subunit 1 gene in mitochondrial DNA and sequenced to discriminate between species of this genus (Appendix).

Consequently, we identified 168 (88.9%) *Anisakis simplex* sensu stricto larvae, 10 (5.3%) *A. pegreffii* larvae, and 11 (5.8%) *Pseudoterranova azarasi* larvae (Table 2). These findings represent perfect sequence identity with the respective species, regardless of the targets. We deposited the obtained sequences in GenBank under accession nos. LC684518–21 (Appendix Table 1).

**Table 2:** Molecular identification of anisakid larvae isolated from patients, Japan, 2018–2019

Species, isolation site	Symptomatic cases, no.			Asymptomatic cases, no.			Total cases, no.			Overall, no. (%)	
	Patients	Larvae		Patients	Larvae		Patients	Larvae		Patients	Larvae
*Anisakis simplex* sensu stricto										160 (88.4)	168 (88.9)
Stomach	139	146*†		18	19‡§		157	165			
Intestine	0	0		3	3‡		3	3			
*Anisakis pegreffii*										10 (5.5)	10 (5.3)
Stomach	9	9*		0	0		9	9			
Intestine	0	0		1	1‡		1	1			
*Pseudoterranova azarasi*										11 (6.1)	11 (5.8)
Oral cavity	0	0		4	4¶		4	4			
Stomach	6	6*#		1	1‡		7	7			
Total	154	161		27	28		181	189		181 (100)	189 (100)

*Abdominal pain. †Two larvae were isolated from 5 patients and 3 larvae were isolated from 1 patient. ‡Larvae were incidentally detected endoscopically during health check-ups or routine follow-up cancer screening. §Two larvae were isolated from 1 patient. ¶*P. azarasi* larvae were spontaneously expelled from the mouth. #Of 6 *P. azarasi* patients, 1 showed urticaria in addition to abdominal pain.

## Conclusions

A recent study estimated 7,700–8,320 annual anisakiasis cases in Spain, a country with a high incidence of anisakiasis in Europe (*10*). Although our survey methods differed, we demonstrated that Japan had an average of 19,737 anisakiasis cases per year during 2018–2019. As preventive measures, the government of Japan has repeatedly instructed local establishments (e.g., restaurants, fish mongers, and grocery stores) and consumers to freeze seafood at −20°C for at least 24 hours before consuming it raw or to remove anisakid nematodes during cooking. The Ministry of Health, Labour and Welfare of Japan provides web-based food poisoning statistics with information regarding fish species reported by anisakiasis patients and preparation procedures associated with infections in Japanese to help consumers and fishmongers avoid anisakiasis.

In Japan, *A. simplex* s.s. nematodes are responsible for the highest incidence of anisakiasis, whereas the species *A. pegreffii* is the leading cause of anisakiasis in Europe and South Korea (*2*). *A. simplex* s.s. nematodes penetrate the muscles of various fish species at a higher rate than *A. pegreffii* (*11*), which could partly explain the smaller proportion of *A. pegreffii* anisakiasis cases in Japan because *A. pegreffii* nematodes are often removed with fish viscera during the preparation of sushi and sashimi. Furthermore, fish habitat can corroborate the difference in predominant anisakid nematode species between South Korea and Japan; *A. simplex* s.s.–carrying fish are predominant in the Pacific side of Japan, whereas *A. pegreffii*–carrying fish are more common in the Sea of Japan and the East China Sea, located between South Korea and Japan (*11*) (Figure 1).

**Figure 2:**
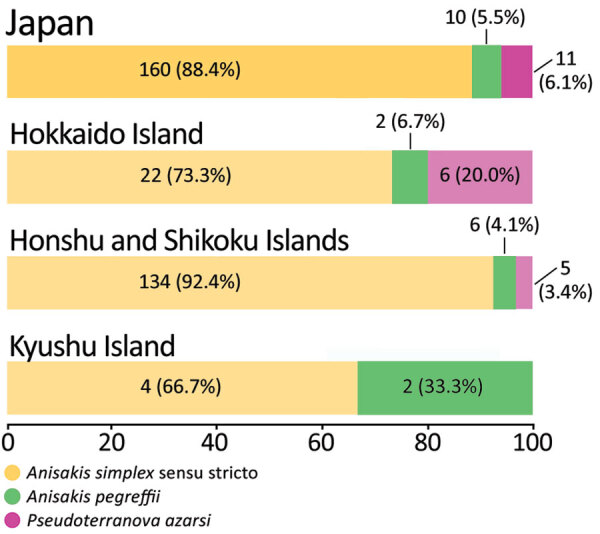
Analyzed number and percentage of anisakiasis patients and causative species, Japan, 2018–2019. One third (2/6) of patients in the Kyushu Island had *Anisakis pegreffii* infections. *A. pegreffii*–carrying fish are predominant in the Sea of Japan and the East China Sea, located between South Korea and Japan (*11*). Over 50% (6/11) of the patients with *Pseudoterranova azarasi* infection were from Hokkaido.

In this study, we identified 11 *P. azarasi* larvae (5.8%) from 11 patients, 6 of whom lived in Hokkaido, the northernmost insular prefecture of Japan, where cold water fish, such as Pacific cod (*Gadus macrocephalus*), are commonly consumed (Figures 1,2) (*12*). Although patients have been reported to orally expel *Pseudoterranova* larvae that have developed to the fourth stage (*13*), most (7/11 cases) *P. azarasi* larvae detected in this study were in the stomach.

Anisakid larvae were isolated from the stomach or intestines of 177 patients, 23 (13%) of whom were asymptomatic. These asymptomatic cases were found incidentally during health check-ups or routine follow-up cancer screening. Some studies have already reported asymptomatic gastrointestinal cases (*14*,*15*), but our findings revealed a varied and greater number of anisakid species associated with asymptomatic infections: *A. simplex* s.s. (91.3%), *A. pegreffii* (4.3%), and *P. azarasi* (4.3%). One urticaria case was associated with symptomatic *P. azarasi* gastric infection. Numerous cases of *Anisakis* allergy, including cases of urticaria, angioedema, and anaphylaxis, have been reported in Europe (*10*). In healthcare claims from Japan, *Anisakis* allergy was not registered as a disease name until 2021. Thus, the database did not include anisakiasis patients with allergic symptoms alone, which is a limitation of this study.

In conclusion, *Anisakis* infection is currently drawing more attention in Japan. Elucidating the immunopathological mechanisms behind asymptomatic and symptomatic anisakiasis is imperative and can provide insights into the pathogenesis of gastrointestinal anisakiasis. 

**Appendix.** Additional information on anisakiasis annual incidence and causative species, Japan, 2018–2019.
