# Supplementary material for: Anisakiasis Annual Incidence and Causative Species, Japan, 2018–2019
Source: Emerg Infect Dis. 2022 Oct;28(10):2105–8. doi: 10.3201/eid2810.220627 (PMC9514333; doi:10.3201/eid2810.220627)
Supplement: Supplementary file 1 — Appendix. Additional information on anisakiasis annual incidence and causative species, Japan, 2018–2019. [file 22-0627-Techapp-s1.pdf]

Article DOI: https://doi.org/10.3201/eid2810.220627

Anisakiasis Annual Incidence and Causative Species, Japan, 2018–2019

Appendix

PCR Amplification and Sequencing

The PCR assay contained a final volume of 50 µL: 5 µL of template DNA, 0.5 µL of Phusion High-Fidelity DNA Polymerase 2.5 U (Thermo Fisher Scientific, https://www.thermofisher.com), 1.25 µL each of primer (20 µmol/L) (Appendix Table 2), 4 µL of dNTP (2.5 mmol/L), and 10 µL of 5× Phusion HF buffer. Amplification was performed by using a Takara PCR Thermal Cycler Dice Gradient thermal cycler (TaKaRa Bio, Inc., http://www.takara-bio.com) with an initial denaturation step at 98°C for 30 s. PCR amplifications for the internal transcribed spacer 1 (ITS1) region were conducted, then 30 cycles of denaturing at 98°C for 10 s, annealing at 55°C for 10 s, and extension at 72°C for 15 s; amplification of the portion of the NADH dehydrogenase subunit 1 gene was performed for 34 cycles at 98°C for 10 s, at 46°C for 30 s, and at 72°C for 1 min. A final extension was performed at 72°C for 7 min for the ITS1 region and 10 min for the NADH gene. Amplicons were sequenced by using the corresponding primers. The dye terminator method was performed by using fluorescently-labeled di-deoxynucleotide triphosphates with the BigDye Terminator version 3.1 Cycle Sequencing Kit (Thermo Fisher Scientific) in accordance with the manufacturer's instructions. Nucleotide sequences were determined by using a 3730xl DNA Analyzer (Thermo Fisher Scientific), and sequence alignments were analyzed by using GENETYX-Win version 13 (GENETYX Co., https://www.genetyx.co.jp).

Appendix Table 1. GenBank accession numbers for anisakid larvae obtained from patients, Japan, 2018–2019*

Species	Target region	Accession no.
Anisakis simplex sensu stricto	ITS1	LC684518
Anisakis pegreffii	ITS1	LC684519
Pseudoterranova azarasi	ITS1	LC684520
	NADH	LC684521

*ITS1, internal transcribed spacer 1 region; NADH, NADH dehydrogenase subunit 1 gene.

Appendix Table 2. Primers used to sequence *Anisakis* parasites obtained from patients, Japan, 2018–2019*

Target	Forward primer	Reverse primer
ITS1	AniT1F2: GTTGAACAACGGTGACCAATTTGGC	AniT1R3: GTACAAATCTTGGCGGTGGATCACTC
NADH	TerNADH-SF6: GCTTGTTAGTGGTTAYAATGTAGAGTAYTC	TerNADH-SR1: CCTAGAAAAATCAAAGAAACAGGCAACAAC

*ITS1, internal transcribed spacer 1 region; NADH, NADH dehydrogenase subunit 1 gene.
